# Supplementary material for: The common house spider, Parasteatoda tepidariorum, maintains silk gene expression on sub-optimal diet
Source: PLoS One. 2020 Dec 9;15(12):e0237286. doi: 10.1371/journal.pone.0237286 (PMC7725297; doi:10.1371/journal.pone.0237286)
Supplement: S2 File — (DOCX) [file pone.0237286.s002.docx]

**S1 Fig. Standard curves used to estimate absolute transcript abundance for three spidroin genes.** Plasmids containing cDNAs for each of the four genes were serially diluted and amplified in qPCR reactions alongside individual spider cDNAs. See Supplementary File 1 for raw Cq values and calculation of absolute abundance using these standard curves.


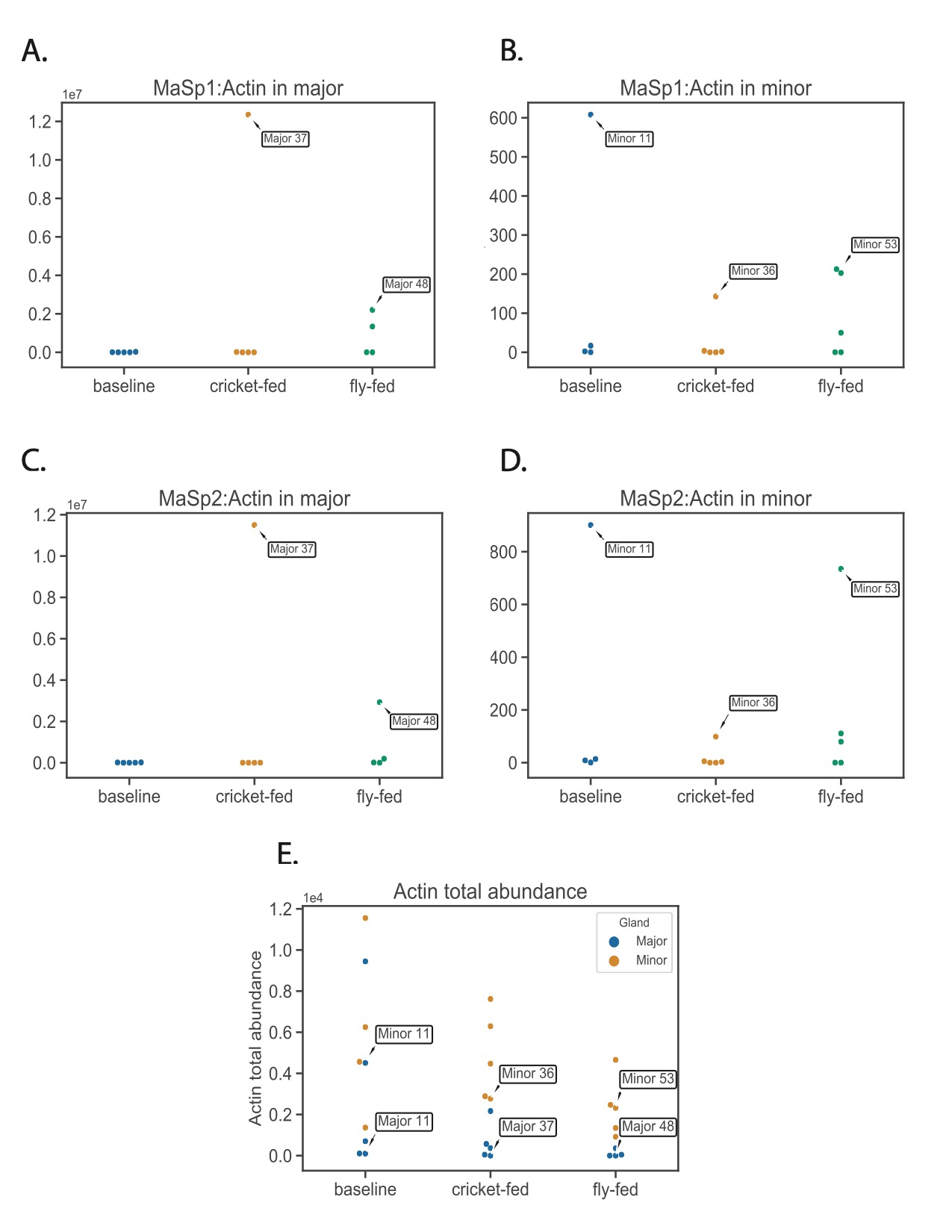


S2 Fig. **Relative abundance in the two glands demonstrated by swarm plots.** Outliers are labeled (a) *MaSp1/Actin* in major ampullate gland (b) *MaSp1/Actin* in minor ampullate gland (c) *MaSp2/Actin* in major ampullate gland (d) *MaSp2/Actin* in minor ampullate gland (e) *Actin* total abundance.

S3 Fig. **Relative and total abundance in the two glands demonstrated by swarm plots.** Outliers are labeled (a) *Actin* total abundance (b) *MaSp1/MaSp2* in minor ampullate gland (c) *MaSp1* total abundance in major ampullate gland (d) *MaSp1* total abundance in minor ampullate gland (e) *MaSp2* total abundance in major ampullate gland (f) *MaSp2* total abundance in minor ampullate gland.
